# Rapid Detection of Peste des Petits Ruminants Virus (PPRV) Nucleic Acid Using a Novel Low-Cost Reverse Transcription Loop-Mediated Isothermal Amplification (RT-LAMP) Assay for Future Use in Nascent PPR Eradication Programme

**DOI:** 10.3390/v11080699

**Published:** 2019-07-31

**Authors:** Mana Mahapatra, Emma Howson, Veronica Fowler, Carrie Batten, John Flannery, Muneeswaran Selvaraj, Satya Parida

**Affiliations:** The Pirbright Institute, Ash Road, Pirbright, Woking, Surrey GU24 0NF, UK

**Keywords:** RT-LAMP, PPRV, rapid detection, pen side, diagnostics, eradication programme

## Abstract

Peste des petits ruminants (PPR) is a disease of small ruminants caused by peste des petits ruminants virus (PPRV), and is endemic in Asia, the Middle East and Africa. Effective control combines the application of early warning systems, accurate laboratory diagnosis and reporting, animal movement restrictions, suitable vaccination and surveillance programs, and the coordination of all these measures by efficient veterinary services. Molecular assays, including conventional reverse transcription-polymerase chain reaction (RT-PCR) and real-time RT-PCR (RT-qPCR) have improved the sensitivity and rapidity of diagnosing PPR. However, currently these assays are only performed within laboratory settings; therefore, the development of field diagnostics for PPR would improve the fast implementation of control policies, particularly when PPR has been targeted to be eradicated by 2030. Loop-mediated isothermal amplification (LAMP) assays are simple to use, rapid, and have sensitivity and specificity within the range of RT-qPCR; and can be performed in the field using disposable consumables and portable equipment. This study describes the development of a novel RT-LAMP assay for the detection of PPRV nucleic acid by targeting the N-protein gene. The RT-LAMP assay was evaluated using cell culture propagated PPRVs, field samples from clinically infected animals and samples from experimentally infected animals encompassing all four lineages (I-IV) of PPRV. The test displayed 100% concordance with RT-qPCR when considering an RT-qPCR cut-off value of C_T_ >40. Further, the RT-LAMP assay was evaluated using experimental and outbreak samples without prior RNA extraction making it more time and cost-effective. This assay provides a solution for a pen-side, rapid and inexpensive PPR diagnostic for use in the field in nascent PPR eradication programme.

## 1. Introduction

Peste des petits ruminants (PPR) is a highly contagious and economically important disease of small ruminants caused by PPR virus (PPRV) of the genus *Morbillivirus* in the family *Paramyxoviridae*. Cattle and buffalo are sub-clinically infected; in addition, a large number of wild species within the order *Artiodactyla* are also affected by PPRV [1]. PPR is endemic in large parts of Africa, the Middle East and Asia, and is still spreading globally, with emergence notably reported in China, Georgia, Mongolia, and most recently within the European Union in Bulgaria [1,2]. PPRV contains a single-stranded negative-sense RNA genome of about 16 kb, organised into six transcription units encoding six structural proteins in the order 3’-N-P-M-F-H-L-5’. In addition, two non-structural proteins, C and V are translated from the P-gene open reading frame (ORF) [3]. Though serologically PPRV exists as a single serotype, genetically it is divided into four (I-IV) distinct lineages [4]. Following the successful eradication of rinderpest, PPR is now target for global eradication by 2030. The eradication program would benefit from new diagnostic tools, particularly field tests to enable detection of viral nucleic acid at the pen side.

Laboratory confirmation of clinical suspicion is made by detection of PPRV in blood, swabs (nasal/ocular/oral) or post-mortem tissues using classical virus isolation (VI), agar gel immunodiffusion or immunocapture ELISA techniques. However, these methods have now been superseded by the adoption of more rapid, sensitive and accurate molecular diagnostic techniques such as reverse transcription-polymerase chain reaction (RT-PCR) based on amplification of parts of either nucleocapsid (N) or fusion (F) protein gene [5,6]. These RT-PCR assays are widely used in most PPR diagnostic laboratories. In addition, few real-time RT-PCR (RT-qPCR) techniques [7,8,9] targeting the nucleocapsid (N) protein gene are also available. Whilst current RT-qPCR methods are accurate, rapid and sensitive, they are required to be performed in laboratory settings and samples must be transported under the appropriate conditions from field to the laboratory that can delay the confirmation of suspicion of PPRV infection, and subsequent implementation of adequate control measures. In addition, sometimes the viral antigen/RNA deteriorates during the transport process making diagnosis difficult. Further, these molecular assays can be time-consuming, labour intensive and expensive especially in case of RT-qPCR. The development of rapid field diagnostic assays that can be used at the point of sample collection may help to overcome these difficulties. 

Loop-mediated isothermal amplification (LAMP) is an isothermal, autocyling, strand-displacement DNA amplification technique [10] that can be performed at a constant temperature. Numerous LAMP assays have been developed for human, plant and veterinary pathogens including paramyxoviruses [11,12,13,14,15,16,17]. In case of PPRV, an RT-LAMP assay based on the M-protein gene [16] and another based on the N-protein gene [11] have been developed; however, they are not known to be extensively used in the field. The present study describes the development and performance of a novel RT-LAMP assay for semi-quantitative detection of PPRV nucleic acid targeting the highly conserved central region of the N-protein gene, benchmarked against one of the RT-qPCR assays [9]. Further, the assay has been evaluated with known clinically positive samples (from experimentally and naturally infected animals) without prior RNA extraction which is time and cost-effective. The RT-LAMP assay developed in this study has a field-based platform which makes it more accessible for field use.

## 2. Materials and Methods

### 2.1. Ethics

All clinical samples from experimentally infected animals were archival samples from previous animal experiment studies that were conducted according to the UK Home Office regulations and after approval by The Pirbright Institute (TPI) Animal Welfare and Ethical Review Board (AWERB), Pirbright, UK. Similarly, all clinical samples from the field used in this study were archival samples that were collected from endemic countries under previous projects. 

### 2.2. Viruses and Clinical Samples for RNA Extraction and Laboratory Evaluation by RT-LAMP

Eight PPRVs encompassing four lineages (I-IV) were used in this study (Table 1). These viruses were grown on VeroDogSLam (VDS) cells and clarified supernatant from infected cells was used in this study. The lineage IV strain Morocco/2008 was used as the reference strain in this study. The outbreak samples (blood, milk, ocular swab, nasal swab and tissues) were collected from four PPR endemic countries such as Algeria (*n* = 8), Tanzania (*n* = 12), Israel (*n* = 3) and Bangladesh (*n* = 4) [18,19,20,21]. All the field samples were of goat origin except for three ocular and three nasal swabs (samples 5–7 and 10–12 in Table 1) that were collected from sheep. In addition, clinical materials such as a nasal swab, faecal samples and various tissues collected from goats experimentally infected with PPRV (Morocco/2008 or Ghana/78) at TPI were also included [22]. Furthermore, filter papers impregnated with virus samples (cell culture supernatant) were also included to account for samples that are usually collected in the field as filter paper spots and later transported to the laboratory for diagnosis. Dolphin morbillivirus (DMV), measles virus (MV), foot-and-mouth disease virus (FMDV, serotype O and A) and swine vesicular disease virus (SVDV) were used for specificity testing.

### 2.3. RNA Extraction

Total RNA was extracted from PPRV infected cell culture supernatant, EDTA blood and animal tissues using TRIzol reagent (Invitrogen, Carlsbad, CA, USA) as described previously [19]. In addition, total RNA was also extracted from milk samples [20]; ocular and nasal swabs collected from sheep and goats in Tanzania [18]) and nasal swabs and faecal samples from experimentally-infected animals [22]. The filter papers impregnated with PPRV were placed in a 1.5 mL microcentrifuge tube containing 200 μL of RNase-free water and incubated at room temperature for ~10 min before being centrifuged at 3000× *g* for 5 min. After centrifugation, the RNase-free water containing virus was transferred to a sterile microcentrifuge tube and total RNA extracted using the Qiagen RNeasy Mini kit (Qiagen, Manchester, UK) according to the manufacturer’s instructions. The RNA was stored at −80 °C as single-use aliquots until tested.

### 2.4. LAMP Primer Design 

Twenty-seven PPRV genome sequences (Accession numbers: KP789375, EU267273, HQ197753, KM212177, X74443, EU267274, KJ466104, KR781451, KJ867540, KJ867543, KM463083, KJ867544, KC594074, JX217850, KM091959, FJ905304, JF939201, KM089832, KM089831, KJ867541, KM089830, KT270355, KR261605, KR140086, KF727981, NC_006383, AJ849636), representing all four lineages were obtained from GenBank. Sequences were aligned in BioEdit (Version 7.0.5.3) from which a highly conserved region was selected in the coding region of the N-gene (nucleotides 108–1685: KC594074). LAMP primers were designed from a consensus sequence of this region using LAMP Designer (OptiGene Ltd., Horsham, UK). A total of three sets of primers were designed to target the highly conserved central region (nucleotide position 800 to 1100) within the N-protein gene and following initial screening one set of primers were taken forward for further analysis (Table 2).

### 2.5. Reverse Transcription Loop-Mediated Isothermal Amplification (RT-LAMP)

RT-LAMP was performed in a total reaction mixture of 25 µL containing 15 µL isothermal master mix ISO-001 (OptiGene Ltd., Horsham, UK), optimised primer concentrations as per Table 2, 2 U AMV reverse transcriptase (New England Biolabs), 5 µL template RNA and made up to volume with nuclease-free water. RT-LAMP reactions were incubated at 65 °C for 60 min on either a Stratagene Mx3005p (Agilent Technologies, Stockport, UK) or Genie II (OptiGene Ltd.). For positive RT-LAMP reactions, the time to positivity (T_p_) was defined when reactions reached a fluorescence threshold increase of δR 1000. 

To confirm that amplicons were PPRV-specific, annealing analysis was performed on RT-LAMP products using the Genie II (OptiGene Ltd.). LAMP products were heated to 98 °C for 1 min, then cooled to 80 °C (ramping at 0.05 °C/s). Anneal temperature (T_a_) calculations were automated using Genie Explorer v0.2.1.1 software (OptiGene Ltd.). Samples were considered positive if amplification had occurred and the LAMP product annealed in the PPRV amplicon-specific temperature range of 83.1–85.1 °C (mean T_a_ 84.1 °C +/- 1 °C of 41 PPRV positive RT-LAMP reactions).

### 2.6. RNA Standards and Dilution Series used to Determine the Analytical Sensitivity of the RT-LAMP Assay

The RNA standard for determining the analytical sensitivity of the PPRV RT-LAMP assay was prepared as previously described [22]. Ten-fold serial dilution series (10^8^–10^1^) of the *in vitro* transcribed PPRV RNA standards were prepared in nuclease-free water-containing carrier RNA (1 µg mL^−1^) and were tested in both the RT-LAMP and RT-qPCR assay to determine the analytical sensitivity. In addition, ten-fold dilution series (10^−1^ to 10^−7^) of the RNA extracted from PPRV strains representing four different lineages (I-IV) were also used to determine the analytical sensitivity of the assay. 

Diagnostic sensitivity was determined using RNA extracted from a total of 54 clinical samples, representing samples from four countries and eight cell culture grown PPRVs (Table 1). Diagnostic specificity was assessed using RNA extracted from four different viruses, two morbilliviruses (MV and DMV), and two viruses (FMDV and SVDV) that cause vesicular disease in animals and are important for differential diagnosis.

### 2.7. Real-Time Reverse-Transcription-Polymerase Chain Reaction (RT-qPCR) 

RT-qPCR was performed as described previously [9] with minor modifications. Briefly, the fluorogenic RT-qPCR was performed in a final volume of 25 μL using a commercial kit Superscript III Platinum^®^ one step qRT-PCR system kit (Invitrogen) on an Applied Biosystems 7500 Fast real-time PCR instrument (Applied Biosystems, Paisley, UK). Cycle threshold (C_T_) values below the cut-off value (C_T_ < 40) were considered positive. 

### 2.8. Sample Preparation for RT-LAMP without RNA Extraction

A total of 11 swab samples (nasal (*n* = 5), eye (*n* = 2) and saliva (*n* = 4)) collected from three goats experimentally infected with PPRV (Ghana/78) [22] and one nasal swab (of goat origin) from field outbreak in Tanzania were selected from our archive stock for this purpose. Following collection, the virus on the swabs was eluted using 2 mL cell culture media (DMEM) and stored at −80 °C. All these swab extracts had been stored at −80 °C for at least two years. In addition, five cell culture supernatants containing PPRVs from all four lineages were also included (Table 4a,b). For testing in the RT-LAMP assay the above swab extracts or freshly prepared cell culture supernatants (comprising of all four lineages of PPRV) were diluted 1:10 using nuclease-free water, and 5 uL of the diluted sample was added to the RT-LAMP reaction mix. 

All samples were run in duplicate in all assays carried out in this study and the samples showing positive in only one well were repeat-tested for confirmation. The average value of two wells was used for subsequent analysis. 

### 2.9. Statistical Analysis

GraphPad prism was used for data analysis and also for generation of the graphs. For all analysis, the RT-qPCR was used as the gold standard.

## 3. Results

### 3.1. Optimisation of the RT-LAMP Assay

Optimisation of the RT-LAMP assay was conducted by examining four different primer concentrations, using RNA derived from PPRV/Morocco/2008 strain (Lineage IV). Out of two enzyme concentrations (2 or 0.2 U of AMV reverse transcriptase) tested; reactions with 2 U of enzyme performed the best. Similarly, out of four different primer concentrations, primer dilution B (containing 1.6 µM FIP/BIP; 0.8 µM Floop/Bloop; 0.2 µM F3/B3) performed the best, reaching an average T_p_ at 20.4 min (Table 3). All the four lineages of PPRV (cell culture supernatant) were detected at these primer and enzyme concentrations, and no non-specific amplification was observed. Therefore, this set was subsequently taken forward for evaluating further samples (involving all four lineages). 

### 3.2. Analytical Sensitivity

**Figure 1:**
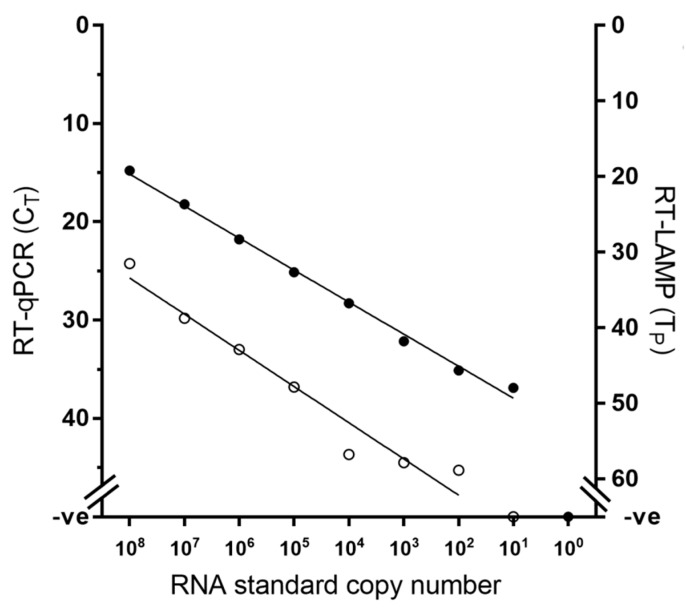
Comparative analytical sensitivity of peste des petits ruminants virus RT-LAMP (open circles) and the gold standard RT-qPCR (closed circles) using in vitro transcribed RNA standards.

**Figure 2:**
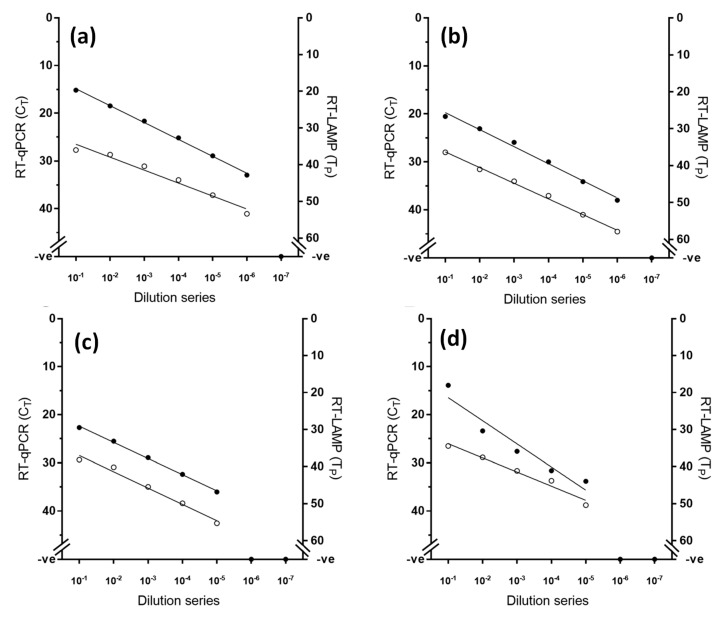
Comparative analytical sensitivity of peste des petits ruminants virus RT-LAMP (open circles) and the gold standard RT-qPCR (closed circles) for four different lineages of PPRV (**a**) lineage I (PPRV/Ivory Coast), (**b**) lineage II (PPRV/Nigeria/76/1), (**c**) lineage III (PPRV/IBRI-Oman) and (**d**) lineage IV (PPRV/Morocco/2008).

Using an established RNA standard [22], the limit of detection of the RT-LAMP assay was defined at 10^2^ RNA copies, while the gold standard RT-qPCR exhibited higher analytical sensitivity than RT-LAMP by one decimal dilution (Figure 1). However, when RNA from viral samples was used, RT-LAMP achieved equivalent analytical sensitivity to the RT-qPCR across the four lineages tested, consistently detecting the same dilution factor (Figure 2).

### 3.3. Diagnostic Sensitivity and Specificity

**Figure 3:**
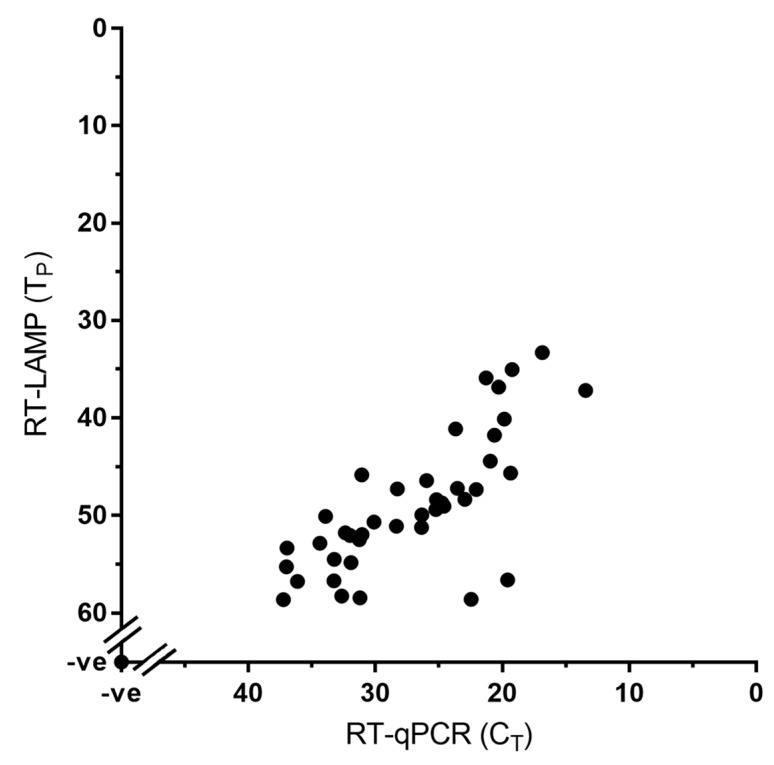
Diagnostic sensitivity of the RT-LAMP assay showing 100% concordance with the RT-qPCR assay. A total of 68 samples were used in this assay; the 41 positive samples (33 clinical samples and eight cell culture grown PPRVs) are shown in the graph whereas the 27 negative samples are represented by a single dot at the intercept of the x- and y-axis.

Both the RT-LAMP and RT-qPCR assay were carried out for the detection of PPRV nucleic acid using a total of 68 samples out of which 54 are clinical samples and eight are cell culture grown PPRVs (Table 1). Of these 54 clinical samples 33 samples were positive for PPRV whereas the remaining 21 samples were negative (showing no T_p_ or T_a_) indicating the diagnostic sensitivity of the RT-LAMP assay to be equivalent to that of the RT-qPCR, with 100% concordance between the two tests (Figure 3; Table 1).

The specificity of the assay was determined by evaluating the cross-reactivity of the assay with different strains of morbillivirus. The PPRV-specific RT-LAMP primer set demonstrated a high degree of specificity for PPRV. All eight PPRV isolates were amplified by this RT-LAMP assay whereas the RNA extracted from five viruses (two closely related morbilliviruses, measles and DMV; and three vesicular disease viruses (FMDV, serotype O and A, and SVDV) yielded negative results (Table 1).

### 3.4. Evaluation of RT-LAMP Assay Using Samples without prior RNA Extraction

Transitioning molecular tests into formats appropriate for field-use requires simplification of the sample preparation process, with RT-LAMP compatible for use on samples directly. Therefore, cell culture supernatants containing PPRV/Morocco/2008 was serially diluted in nuclease-free water and tested by both RT-LAMP and RT-qPCR assays to find out their suitability to be used directly, without prior RNA extraction. Amplification was observed in the RT-LAMP assay whereas no amplification was observed when using the gold standard RT-qPCR assay (Table 4a). Furthermore, amplification was observed up to a dilution of 10^−4^ in water (Table 4a).

With the success of detection of PPRV nucleic acid using cell culture propagated virus samples without prior RNA extraction, further samples were evaluated in the RT-LAMP assay (Table 4b). The cell culture supernatants comprising of all four lineages of PPRV were detected in the test. A total of 11 clinical samples (eight known positives and three known negatives) from experimentally infected animals and one known positive sample from a field outbreak were selected from our archive stock; the swab elutes of these samples had been stored at −80 °C for at least 2 years. Of the eight known positive samples, six were positive in the RT-LAMP assay without RNA extraction. The one known positive sample from a field outbreak was also found to be positive in the RT-LAMP assay without RNA extraction (Table 4b).

## 4. Discussion

Among the structural proteins, the N-protein is antigenically the most conserved among morbilliviruses and is highly immunogenic [12,23]. Since the N-protein gene is expressed (gradient expression) to a very high level in virus-infected cells, it has been used as an antigen for diagnostic purposes for rabies virus [24], measles virus [25], Newcastle disease virus (NDV) [26] and PPRV [23,27]. Alignment of morbillivirus N-protein amino acid (aa) sequences has defined four regions (I-IV) with varying degree of sequence homology out of which region-III (aa 145−420, equivalent nucleotide position 433-1260 in N-protein gene open reading frame) represents the central highly conserved region [28]. This area has also been targeted in N-protein gene-based diagnostic RT-PCR and RT-qPCR [6,7,9]. In this study, an RT-LAMP assay was developed targeting the highly conserved region-III of the N-protein gene and evaluated using cell culture supernatant containing PPRVs from all four lineages, clinical samples from the field in endemic settings and also from animals experimentally infected with PPRV.

PPR can be confused with several other diseases (FMD, bluetongue, goat/sheep pox, contagious caprine pleuropneumonia etc.) due to the similarity in clinical symptoms [1]. Therefore, in addition to clinical observations, a differential diagnosis must be confirmed by laboratory-based diagnostic techniques. The currently available molecular diagnostic tests that detect viral nucleic acids (RT-PCR, RT-qPCR, RT-LAMP) are rapid and sensitive [29]. However, the efficiency of laboratory diagnosis can be greatly influenced by the type and integrity of the sample received [1,29]. The recommended specimens from live animals are nasal, ocular and oral swabs, blood; dead animals: lymphoid tissues and lung tissue; wildlife: non-invasive samples like faecal material [30]. Further, the best sample for conventional RT-PCR is buffy coat [31]; RT-qPCR: nasal swab and blood [22]. Therefore, in this study, a broad range of known positive samples (blood, tissues, nasal, saliva and ocular swabs, milk and faeces) were included.

The RT-LAMP assay was found to be as sensitive as the RT-qPCR test used in this study on clinical samples and could be used in its current format as a valuable complementary tool to standard, lab-based RT-qPCR assays. Two other PPRV RT-LAMP assays, one based on the M-protein gene and the second based on the N-protein gene have been developed, with equivalent performance to RT-qPCR [11,16]. The M-protein gene based assay currently relies on detection of the amplified product by the naked eye. The use of a portable fluorimeter enables semi-quantitative, real-time and objective detection, which can be performed by a non-skilled user. Furthermore, the ability to perform an anneal-curve at the end of the reaction differentiates between targeted and non-specific amplification providing further confidence in the results. The N-protein gene-based assay had been evaluated using samples from only two lineages (II and IV) of PPRV and the clinical samples comprised of only nasal swabs whereas our assay was evaluated using all the four lineages of PPRV and the clinical samples included a broad range of sample types (nasal, saliva and ocular swab, blood, faeces, milk, tissues etc.). In addition, the existing N-protein gene-based assay uses eight units of enzyme per reaction, four times more than our assay. Addition of more enzyme is known to enhance the overall cost per reaction. Cost-effective assays with similar detection limit are beneficial for the nascent eradication programme in PPR endemic low- and middle-income countries (LMIC). 

Using *in vitro* synthesised RNA standards, the limit of detection of the RT-LAMP assay was found to be 10^2^ RNA copies while that of the gold standard RT-qPCR assay was ten-fold higher. However, when RNA from cell culture propagated virus samples (supernatant) were used, RT-LAMP achieved equivalent analytical sensitivity to the RT-qPCR across the four lineages tested, consistently detecting the same dilution. Samples from clinical cases of PPRV usually have high viral loads, which would fall within the detection range of the PPR RT-LAMP assay. This would allow for the rapid detection of positive samples and defined the RT-LAMP assay as a valuable tool for use during outbreak investigations. 

In case of several other pathogens of veterinary importance, RT-LAMP assays could be carried out directly on the samples without extracting nucleic acids [17,32] that saves time and allows the assay to be rapid and efficient for field application. As such, preliminary investigations were carried out in this study to detect PPRV nucleic acids in the samples without prior requirement for RNA extraction, allowing simple sample preparations, i.e., dilution in nuclease-free water. Amplification was observed in the RT-LAMP assay when cell culture propagated PPRV was diluted in water, whereas no amplification was observed in gold standard RT-qPCR assay. This is not surprising as LAMP chemistry has been consistently reported to show increased tolerance to inhibiting factors that might be present in clinical samples compared to PCR [17,33]. Therefore, the PPRV RT-LAMP assay has an additional benefit of simple sample preparation by removal of the RNA extraction step, increasing its compatibility with field use. Further, outbreak samples from clinically infected animals, samples from experimentally infected animals and cell culture grown PPRVs, encompassing all four lineages (I-IV) were detected in the RT-LAMP assay without prior RNA extraction. However, two known positive swab samples with low viral load were not detected in this assay. This could be due to the effect of long term storage of these samples (more than two years) where RNA might have degraded or due to the effect of diluting the samples (swabs eluted in 2 mL media). Therefore, a large number of samples (swabs and blood) from known clinically infected animals (experimentally infected and from PPR outbreaks) and the dilution factors need to be tested to validate the RT-LAMP assay without prior RNA extraction. This is currently being taken forward in an on-going project in our laboratory. 

In order to facilitate the use of this assay in the field, future work should consider further testing and validation of the removal of nucleic acid extraction steps, the lyophilisation of the assay reagents, and the development of an alternative visualisation strategy such as objective detection of amplified product using molecular lateral flow devices [17]. It would be appropriate to extend the development of this RT-LAMP assay to introduce the aforementioned modifications and testing the performance of the assay directly in the field during real outbreak investigations. 

In conclusion, we have developed a novel, rapid and cost-effective PPRV RT-LAMP assay with 100% specificity and sensitivity in comparison to existing gold-standard RT-qPCR assay that could be applied in the field and could improve early warning systems and control of PPR.

## Figures and Tables

**Table 1:** Virus isolates and clinical samples used for the development and evaluation of the PPRV RT-LAMP assay. Samples 1–27 were collected from the field whereas samples 28–51 were collected from the animals experimentally infected with PPRV (Morocco/2008) at The Pirbright Institute (TPI). Samples 1–12 were collected from Tanzania, 13–20 from Algeria, 21–24 from Bangladesh and 25–27 from Israel. The filter paper samples (52–54) were prepared in the lab. Samples with an asterisk are of sheep origin. C_T_: Cycle threshold values, T_P_: Time to positivity, T_a_: Anneal temperature. The lineages of the PPR viruses in sample numbers 55–62 are shown in parenthesis under sample details.

Sample No.	Sample Details	RT-qPCR Mean C_T_	RT-LAMP Mean T_p_	RT-LAMP Mean T_a_
1	Occular swab-G4	no C_T_	No T_p_	No T_a_
2	Occular swab-G10	22.96	48.32	84.25
3	Occular swab-G11	36.96	53.29	84.06
4	Occular swab-G16	26.37	51.204	84.08
5	Occular swab-S14*	no C_T_	No T_p_	No T_a_
6	Occular swab-S19*	no C_T_	No T_p_	No T_a_
7	Occular swab-S20*	no C_T_	No T_p_	No T_a_
8	Nasal swab-goat-1	37	55.24	84.11
9	Nasal swab-goat-2	32	52.03	84
10	Nasal swab-sheep-1*	34.36	52.82	84.06
11	Nasal swab-sheep-2*	no C_T_	No T_p_	No T_a_
12	Nasal swab-sheep-5*	31.04	51.93	84.1
13	Farm 1-blood 1	25.23	49.35	84
14	Farm 1-blood 2	22.05	47.31	84.13
15	Farm 1-blood 3	19.36	45.63	84.1
16	Farm 1-blood 4	no C_T_	No T_p_	No T_a_
17	Farm 1-blood 5	26.34	49.91	84.17
18	Farm 1-blood 6	33.27	56.65	84.05
19	Farm 1-blood 7	20.95	44.39	84.11
20	Farm 1-blood 8	31.27	52.43	84.09
21	Milk-B52/Chuadanga/2015	24.76	48.69	84
22	Milk-B53/Savara/2015	30.1	50.63	84.06
23	Milk-B18/Nihkanchari/2015	24.61	49.03	84.1
24	Milk-B19/Nihkanchari/2015	23.54	47.17	84
25	Field lung tissue-1	20.62	41.73	84.2
26	Field lung tissue-2	28.26	47.23	84.15
27	Field lung tissue-3	no C_T_	No T_p_	No T_a_
28	Faecal sample-1	noC_T_t	No T_p_	No T_a_
29	Faecal sample-2	no C_T_	No T_p_	No T_a_
30	Faecal sample-3	no C_T_	No T_p_	No T_a_
31	Faecal sample-4	19.6	56.575	84.1
32	Faecal sample-5	22.45	58.565	83.94
33	Nasal swab-1	no C_T_	No T_p_	No T_a_
34	Nasal swab-2	32.65	58:21:00	83.99
35	Nasal swab-3	31.22	58:41:00	84.06
36	Nasal swab-4	25.18	48.35.00	84
37	Nasal swab-5	28.35	51:06:00	84.19
38	Lymp node—bronchial-1	noC_T_	No T_p_	No T_a_
39	Lymp node—bronchial-2	no C_T_	No T_p_	No T_a_
40	Lymp node—bronchial-3	no C_T_	No T_p_	No T_a_
41	Lymp node—bronchial-4	33.24	54.45	84.18
42	Lymp node—bronchial-5	no C_T_	No T_p_	No T_a_
43	Lymp node—bronchial-6	no C_T_	No T_p_	No T_a_
44	Spleen	no C_T_	No T_p_	No T_a_
45	Lymp node—mandibular	33.92	50.05	84.09
46	Lymp node—prescapular	no C_T_	No T_p_	No T_a_
47	Tonsil—pharyngeal	31.08	45.8	84.05
48	Tonsil—palatine	32.37	51.73	84.08
49	Ileum	no C_T_	No T_p_	No T_a_
50	Colon	no C_T_	No T_p_	No T_a_
51	Rectum	no C_T_	No T_p_	No T_a_
52	Filter paper impregnated with virus-1	36.12	56.73	83.99
53	Filter paper impregnated with virus-2	37.23	58.58	83.94
54	Filter paper impregnated with virus-3	31.92	54.8	84.08
55	PPRV/Nigeria/75/1 (L-II)	19.24	35.02	84.07
56	PPRV/Nigeria/76/1 (L-II)	23.68	41.09	84.12
57	PPRV/Sungri/96 (L-IV)	13.45	37.15	84.15
58	PPRV/Ivory coast (L-I)	20.30	36.8	84.1
59	PPRV/IBRI-Oman (L-III)	19.84	40.08	84
60	PPRV/Morocco/2008 (L-IV)	16.85	33.28	84.1
61	PPRV/Bangladesh/170/2012 (L-IV)	25.96	46.39	84
62	PPRV/Ghana/78 (L-II)	21.29	35.86	84.1
63	Nuclease-free water (negative control)	no C_T_	No T_p_	No T_a_
64	Dolphin Morbillivirus (DMV)	no C_T_	No T_p_	No T_a_
65	Measles virus (MV)	no C_T_	No T_p_	No T_a_
66	FMDV/O/PanAsia-2	no C_T_	No T_p_	No T_a_
67	FMDV/A/A22/Iraq 24/64	no C_T_	No T_p_	No T_a_
68	Swine Vesicular Disease virus (SVDV)	no C_T_	No T_p_	No T_a_

**Table 2:** Oligonucleotide primers used for RT-LAMP amplification of PPRV; position mapped to GenBank accession: KC594074.

Primer Name	Type	Sequence (5ʹ–3ʹ)	Position	Final Concentration
F3	Forward outer	TCATACTTGACATCAAGAGGAC	814–835	0.2 µM (5 pmol)
B3	Reverse outer	GAGTTCTCTAGAATTACCATGTAGG	1039–1063	0.2 µM (5 pmol)
FIP(F1c+F2)	Forward inner	GTTTCAATACCAAACTTGATAGTAAGGATG	867–884	1.6 µM (40 pmol)
		ATCTGCGACATTGACAAC	914–943	
BIP(B1c+B2)	Reverse inner	GTATCCTGCATTAGGTCTTCACGAG	947–971	1.6 µM (40 pmol)
		CCTAGTTGTTGATACAAGTTCATC	1004–1027	
Floop	Forward Loop	CAAGTCCGGCTTCGACAATA	887–906	0.8 µM (20 pmol)
Bloop	Reverse Loop	GGAATTGTCCACTATAGAATCCCT	980–1003	0.8 µM (20 pmol)

**Table 3:** Effect of RT-LAMP primer concentrations on performance of the assay. T_P_: Time to positivity.

Primer Name	Reaction A		Reaction B		Reaction C		Reaction D	
	Concentration	Average T_P_	Concentration	Average T_P_	Concentration	Average T_P_	Concentration	Average T_P_
F3	0.2 µM	23.32	0.2 µM	20.4	0.2 µM	22.6	0.2 µM	23.78
B3	0.2 µM		0.2 µM		0.2 µM		0.2 µM	
FIP(F1c+F2)	2.0 µM		1.6 µM		1.2 µM		0.8 µM	
BIP(B1c+B2)	2.0 µM		1.6 µM		1.2 µM		0.8 µM	
Floop	1.0 µM		0.8 µM		0.6 µM		0.4 µM	
Bloop	1.0 µM		0.8 µM		0.6 µM		0.4 µM	

**Table:** (**a**)

Sample Details	Dilution	Average T_p_	Average T_a_	C_T_-Values
PPRV/Morocco/2008 diluted in nuclease-free water	10^−1^	46.3	84.25	No C_T_
	10^−2^	46.65	84.25	No C_T_
	10^−3^	52.3	84.2	No C_T_
	10^−4^	56.45	84.3	No C_T_
	10^−5^	No T_p_	No T_a_	No C_T_

**Table:** (**b**)

Sample No.	Sample Details	dpc	Average T_p_	Average T_a_
1	Nasal-G1	0	0	0
2	Nasal-G1	9	38.15	84.4
3	Nasal-G1	10	36.63	84.55
4	Nasal-G2	8	51.15	84.9
5	Nasal-G2	10	0	0
6	Eye-G3	0	0	0
7	Eye-G3	8	51.15	84.9
8	Saliva-G1	0	0	0
9	Saliva-G1	9	29	84.5
10	Saliva-G2	9	30	84.6
11	Saliva-G2	10	0	0
12	IBRI-Oman ( L-III)*	TCS	36.375	84.75
13	Nigeria 75/1 (L-II)*	TCS	25.8	84.35
14	Ivory Coast (L-I)*	TCS	16.07	84.35
15	Sungri/96 ( L-IV)*	TCS	38.08	84.25
16	Nasal- G10**	Field sample	38.3	84.35
